# The potential of regulatory T cell-based therapies for alopecia areata

**DOI:** 10.3389/fimmu.2023.1111547

**Published:** 2023-05-02

**Authors:** Sheng Wan, Wen Xu, Bo Xie, Cuiping Guan, Xiuzu Song

**Affiliations:** ^1^ Department of Dermatology, Hangzhou Third People’s Hospital, Hangzhou Third Hospital Affiliated to Zhejiang Chinese Medical University, Affiliated Hangzhou Dermatology Hospital, Zhejiang University School of Medicine, Hangzhou, China; ^2^ School of Medicine, Zhejiang University, Yuhangtang, Hangzhou, China

**Keywords:** alopecia areata, regulatory T cell, autoimmune disease, immune homeostasis, hair follicle, T_reg_ cell-based therapy, CAR-T_reg_ cell, low-dose IL-2

## Abstract

Cytotoxic T lymphocyte has been a concern for the etiopathogenesis of alopecia areata (AA), some recent evidence suggests that the regulatory T (T_reg_) cell deficiency is also a contributing factor. In the lesional scalp of AA, T_reg_ cells residing in the follicles are impaired, leading to dysregulated local immunity and hair follicle (HF) regeneration disorders. New strategies are emerging to modulate T_reg_ cells’ number and function for autoimmune diseases. There is much interest to boost T_reg_ cells in AA patients to suppress the abnormal autoimmunity of HF and stimulate hair regeneration. With few satisfactory therapeutic regimens available for AA, T_reg_ cell-based therapies could be the way forward. Specifically, CAR-T_reg_ cells and novel formulations of low-dose IL-2 are the alternatives.

## Highlights

▪ T_reg_ cells act as an indispensable component to maintain self-tolerance and immune homeostasis.
▪ Defective T_reg_ cells are a typical feature of almost all autoimmune diseases, including AA.
▪ Skin-resident T_reg_ cells are the HF-IP guardians. They localize to the HFSC niche and promote hair regeneration, which is the basis for T_reg_ cell-based therapies for AA.
▪ Antigen-specific T_reg_ cells could be generated for trials of AA-targeted therapy, and potential autoantigens need to be identified urgently.
▪ Low-dose IL-2 in combination with other treatments is expected to enhance the efficacy of therapy for AA.

## Introduction

It is well acknowledged that alopecia areata (AA), which causes non-scarring hair loss, is a T cell-mediated autoimmune phenomenon affecting approximately 2% of the population (1, 2). Patients with severe AA have poor quality of life and suffer from high levels of anxiety and depression (3). Certain issues cause hair follicle immune privilege (HF-IP) to fall apart, which is thought to be the driver of AA (4). However, the exact pathogenesis of AA remains to be fully characterized. The use of nonspecific immunosuppressants were the mainstay of treatment for the majority of AA patients in the past. Although novel therapies have progressed rapidly in recent years (5), they are still unsatisfactory and new treatment options are essential. New evidence suggests that several other cells have been implicated besides the “central players”, CD8^+^ T cells, such as T-helper (T_H_) 17 cells, natural killer (NK) cells, mast cells, plasmacytoid dendritic cells (pDCs), and regulatory T (T_reg_) cells (6–9). T_reg_ cell is one of the regulatory lymphocyte subsets, maintaining self-tolerance and immune homeostasis of the body. Interestingly, defects in T_reg_ cell function are strongly linked to various autoimmune disorders (10, 11) and AA is no exception (12). Therefore, by enhancing the function of T_reg_ cells, restoration of immune tolerance in patients with autoimmune illnesses has now been achieved (13, 14), but for AA, there are few relevant studies.

The current review focuses on T_reg_ cells and their close association with AA, and specific roles in HF have been discussed with an emphasis on the therapeutic potential of T_reg_ cells. We aim to broaden the horizon of AA research and offer some suggestions for therapeutic use.

## T_reg_ cell biology

Self-tolerance, the state of unresponsiveness to self-tissues (antigens), is pivotal in the field of immunology. The breakdown of this state results in tissue inflammation and autoimmunity. Central tolerance (recessive) and peripheral tolerance (dominant) are the two mechanism categories (15). It is believed that regulatory T cells, in particular, induce and maintain peripheral tolerance.

Early in the 1960s, neonatal mice with thymectomy were found to have wasting syndrome, which closely resembles graft-versus-host reactions (16). Further research suggested the existence of “suppressor T cells” in lymphocytes (17–19), but the immunological field remained skeptical of them (20). Until 1995, a subset of CD4^+^ T cells that constitutively express interleukin-2 (IL-2) receptor α-chain (CD25) were first described by Sakaguchi et al. (21); these CD4^+^CD25^+^ T cells potently repressed the function of conventional T (T_conv_) cells. The concept of regulatory T (T_reg_) cells was thus formally put forward. However, as CD25 is upregulated in all activated T cells, it is thought that CD25^+^ T_reg_ cells are simply T_conv_ in an active state. Finally, the forkhead box P3 (Foxp3) discovery laid the foundation for T_reg_ cell biology (22, 23). This transcription factor is essential for the development, phenotype maintenance, and activity of T_reg_ cells (24–28). Since that, CD4/CD25/Foxp3 have become reliable phenotypic markers to identify T_reg_ cells (in a narrow sense) and this field has been facilitated largely.

### Types of T_reg_ cell

In fact, CD4^+^CD25^+^FOXP3^+^ T_reg_ cells are just one of the classic population of T_reg_ cells. To date, several subsets of T_reg_ cells have been described. However, whether any definitive phenotypic marker exists remains controversial (29, 30). The terms of these subsets are not uniform and oftentimes confusing. A recommendation has been proposed to simplify the nomenclature but limited progress has been made (31). Some researchers classify them according to their developmental origin: thymus-derived T_reg_ (tT_reg_) cells and peripherally derived T_reg_ (pT_reg_) cells (32, 33). Others divide them into natural T_reg_ (nT_reg_) cells and induced T_reg_ (iT_reg_) cells (34, 35), and still, others argue that there are three subgroups of T_reg_ cells, namely, nT_reg_ cells, pT_reg_ cells, and iT_reg_ cells (36, 37). Undoubtedly, both nT_reg_ cells and pT_reg_ cells refer to CD4^+^CD25^+^FOXP3^+^ T_reg_ cells (29, 33), which are naturally occurring T_reg_ cells derived from the thymus as a separate lineage. They are induced by the thymus’s TCR signal and costimulatory molecules (36, 38). These CD4^+^CD25^+^Foxp3^+^ T_reg_ cells constitute 5%–10% of CD4^+^ T cells (34, 39). pT_reg_ cells develop from naïve CD4^+^ T cells in the periphery after contact with antigens and in the presence of specific factors such as transforming growth factor-beta (TGF-β) and IL-2 (29, 40). Some of them circulate through the blood and peripheral lymphoid organs. While others exist in non-lymphoid tissues, these T_reg_ cells are what we commonly call “tissue T_reg_ cell” or “tissue-resident T_reg_ cell” (this notion was advanced in 2009) (41). Moreover, these cells fit the criteria for effector memory cells (CD45RO^+^); thus, they are also named memory T_reg_ cells (42). The iT_reg_ cells could be induced by TGF-β *in vitro* (36, 37). Many researchers conflate the latter two and regard them as “iT_reg_ cells”; hence, it is not easy to understand whether they are referring to the T_reg_ cells generated *in vitro* or *in vivo*.

Regarding the function of these subsets, it was not initially thought that iT_reg_ cells had sufficient suppressive activity compared to nT_reg_ cells (35, 43) due to the loss of Foxp3 expression in iT_reg_ cells, resulting from failing to fully demethylate the T_reg_ cell-specific demethylated region (TSDR). Then, by exploring the plasticity and stability of iT_reg_ cells (34, 40), recent studies have established the position of iT_reg_ cells in immunological tolerance. More details of the types of T_reg_ cells have been summarized (30, 44); no further description will be given here, and they are also not strictly differentiated in this review for the convenience of follow-up discussion.

### Mechanisms of T_reg_ cell-mediated suppression

T_reg_ cell-mediated suppression serves as a vital mechanism for negative regulation of immune-mediated inflammation. There are concise descriptions of the three main categories of mechanisms involved.

**1. Cell–cell contact.** T_reg_ cells constitutively express cytotoxic T lymphocyte-associated protein 4 (CTLA-4). CTLA-4 competes with CD28, a T-cell costimulatory molecule, for CD80/CD86 on antigen-presenting cells (APCs) and downregulates expression of the latter, resulting in the inhibition of T_conv_ cells (45, 46). Furthermore, CTLA-4 upregulates indoleamine 2,3-dioxygenase (IDO), resulting in cell cycle arrest and increased sensitivity to apoptosis in effector T (T_eff_) cells, and dysfunctional APCs (47, 48). Like CTLA-4, lymphocyte activation gene 3 (LAG-3) is highly expressed on the surface of T_reg_ cells, which inhibits the function of dendritic cells (DCs) (49, 50). Moreover, T_reg_ cells could directly induce apoptosis of target cells *via* cell contact, attributing to their release of cytotoxic factors such as granzymes (51, 52).

**2. Secretion of inhibitory cytokines.** T_reg_ cells secrete some inhibitory cytokines such as IL-10, TGF-β, and IL-35, which suppress both T_eff_ cells and APCs. Major histocompatibility complex (MHC) class II molecules of APCs are downregulated in the presence of IL-10 (53). TGF-β may inhibit T cells and (or) maintain Foxp3 expression in T_reg_ cells (54), and IL-35 could reduce T-cell proliferation (55).

**3. Metabolic disruption of T_eff_ cells.** T_reg_ cells scarcely produce IL-2 but consume IL-2 from the surroundings *via* their high-affinity IL-2 receptor, resulting in cytokine deprivation-induced apoptosis of T_eff_ cells (56, 57). Besides that, T_reg_ cells express CD39 and CD73, which generate adenosine and cyclic adenosine monophosphate (cAMP); the former could increase intracellular cAMP of T_eff_ by adenosine receptor 2A, disrupting their metabolism (58, 59).

## T_reg_ cells in alopecia areata

Presumptive T cell-mediated autoimmune illness of the skin with destruction to the hair follicle (HF) is known as alopecia areata (AA). However, the pathobiology of this chronic, relapsing hair-loss disorder is not entirely known. IFN-γ, IL-15, and CD8^+^NKG2D^+^ T cells have long been identified as the core contributors to pathological processes, but numerous studies have shown that they are not the only drivers and several other cell populations may be the new “player”. T_reg_ cells, regulating the immune response and maintaining peripheral tolerance. Thus, an important new frontier is the role of regulatory lymphocytes in maintaining the HF immune privilege (HF-IP). Meanwhile, the discovery of tissue-resident T_reg_ cells will give us a thorough grasp of T_reg_ cells’ roles in HF. Although the mechanism of T_reg_ cell weakening in AA has not been illuminated, it may be an exciting subject for future studies. To avoid redundancy, we will analyze the contributions of T_reg_ cells in AA pathogenesis.

### Impaired T_reg_ cells

Many previous studies have revealed defects in the frequencies and functions of T_reg_ cells in virtually all the common systemic autoimmune disorders (11, 60, 61), but little is known about AA. A genome-wide association study in AA identified several genes controlling the activation and proliferation of T_reg_ cells (62). With the growing appreciation of T_reg_ cells, related articles have been published. Circulating T_reg_ cells from AA patients have been found to have impaired inhibitory activity (63). In other words, T_eff_ cells are relatively dominant. Some researchers proposed that the imbalance between T_H_17 cells and T_reg_ cells is crucial in the pathogenesis (64–66). However, some of the results in the literature are controversial. Two excellent reviews (67, 68) gave an overview of T_reg_ cells in autoimmune skin diseases including AA, vitiligo, psoriasis, and systemic sclerosis. Interestingly, almost all of them were linked to abnormal T_reg_ cell function. Thus, there is a reasonable prospect that it may be a “universal law” in the pathogenesis of the autoimmune disease. This raises a question: why does the imbalance occur? Next, we seek to offer a perspective of T_reg_ cells in lesional HF.

**Figure 1:**
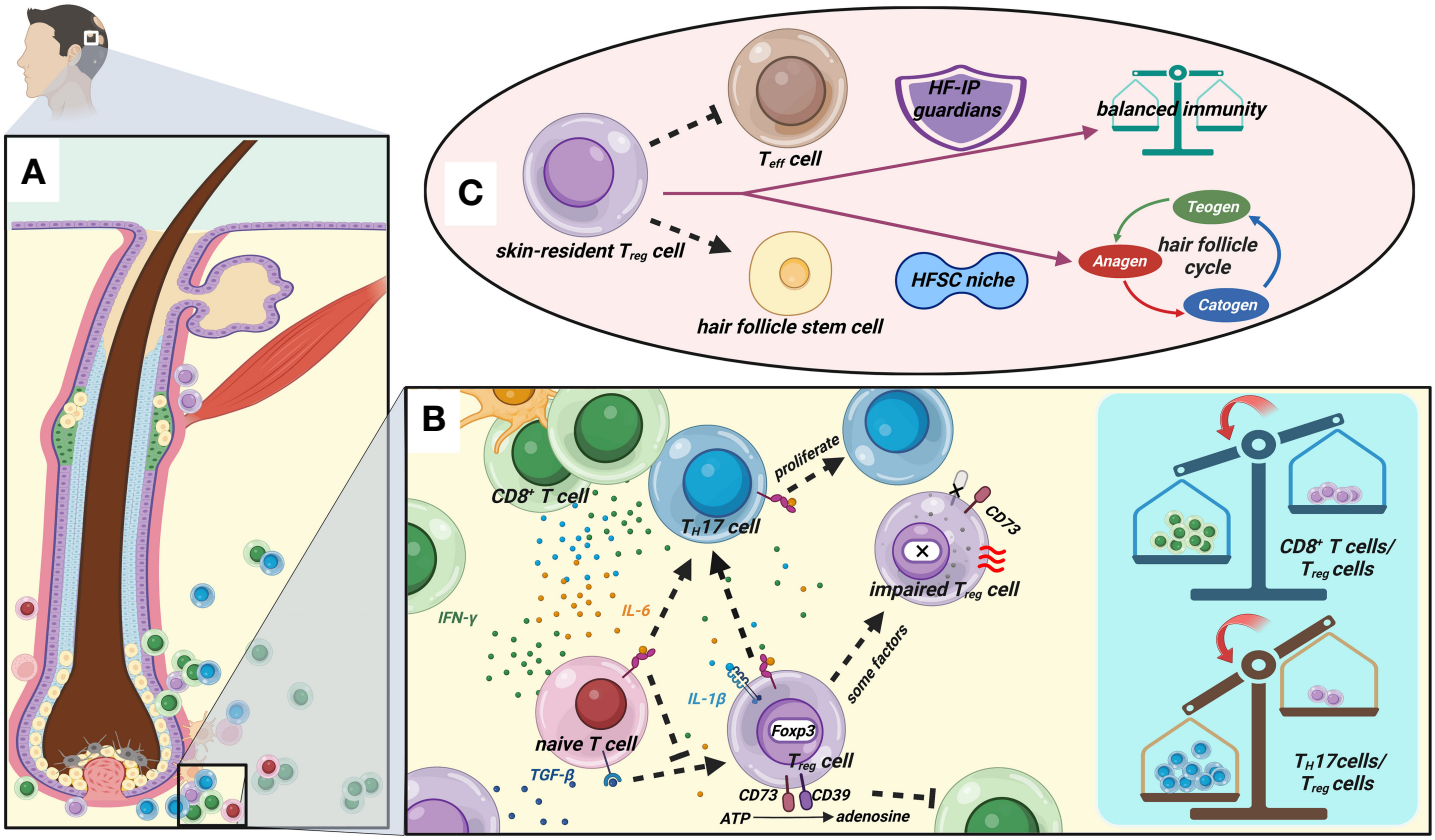
Great importance of T_reg_ cells in AA. **(A)** The anagen HF of AA patients. **(B)** There are tremendous amounts cytokines in the HF after immune attack. IL-6 inhibits the generation of Foxp3^+^ T_reg_ cells induced by TGF-β and induces the differentiation of pathogenic T_H_17 cells. IL-6 and IL-1β could reverse T_reg_ cells’ suppressive function or result in their conversion to T_H_17 cells. Some issues lead to intrinsic defects of T_reg_ cells such as loss of expression of CD39 and Foxp3. Eventually, impairment of T_reg_ cells contributes to the imbalance of immunocyte populations including CD8^+^ T cells/T_reg_ cells and T_H_17 cells/T_reg_ cells. **(C)** Skin-resident T_reg_ cells have dual roles in maintaining immune homeostasis as HF-IP guardians and localizing in the HFSC niche to drive the cyclic growth of HF. (**Figure1** created with BioRender.com).

Gilhar et al. (69) theorized that the IFN-γ “storm” and CD8^+^ T cells might break the delicate balance of immune cells, and that there will be tremendous and complex cytokines in the environment after the HF under immune attack (**Figures 1A, B**). TGF-β and IL-6 together stimulate the development of pathogenic T_H_17 cells from naive T cells, and IL-6 can inhibit the production of Foxp3^+^ T_reg_ cells generated by TGF-β (70). Moreover, due to the plasticity of T_reg_ cells, IL-6 and IL-1β could also reverse their suppressive function or result in their conversion to T_H_17 cells (29, 71). This phenomenon has been demonstrated in some other disorders (72–74). Considering that some reports on AA found that the percentage of circulating T_reg_ cells was normal, T_reg_ cells’ intrinsic defects may be another main reason, such as defects in suppression, survival, or stability. As we mentioned earlier, CD39 is a key component in T_reg_ cell suppressive machinery. However, there was significantly reduced CD39 and HLA-DR expression on circulating T_reg_ cells and HF T_reg_ cells in AA patients, and Hamed et al. (12) speculated that impaired T_reg_ cell function is mainly due to a defect in cell–cell contact and CD39-mediated suppression. The opposite is true in most cancers (75). CD39^+^ T_reg_ cells are strong suppressors of T_H_17 cells and NK cells in antitumor immunity (76, 77). Moreover, Conteduca et al.’s study suggested that Foxp3 and inducible costimulatory ligand (ICOSLG) polymorphisms may predispose to AA by decreasing its mRNA expression (78), which is due to the destruction of the stability of T_reg_ cells. In conclusion, there could be many reasons for defective T_reg_ cell suppression.

### Skin-resident T_reg_ cells: “HF-IP guardians” localize to the HFSC niche?

IP was proposed in the 1940s as a relative and dynamic special status of self-tolerance, which occurs in tissues and organs including the eye, testis, heart valves (79–81), and, of course, HF (82, 83). There are at least two distinct areas of relative IP in HF: anagen hair bulb and the bulge region (4). Mechanisms of HF IP (these two IP areas are slightly different) are thought to downregulate the expression of MHC I and β2-microglobulin (84), and the secretion of immunosuppressive cytokines and neuropeptides (“IP guardians”) such as TGF-β1/2, IL-10, alpha-melanocyte-stimulating hormone (α-MSH), calcitonin gene-related peptide (CGRP), and vasoactive intestinal peptide (VIP) (85–87). Anagen hair follicles’ immune privilege maintaining mechanism collapses in patients with AA, but the cause is a matter of debate (88). Recently, there has been a suspicion that T_reg_ cells may also be a type of key HF-IP guardian. Perifollicular T_reg_ cells create a local immunoinhibitory environment to maintain HF-IP. This peripheral tolerance is critical for HF because some HF-associated antigens are not expressed in the thymus (89).

One of the most important advances in this topic concerns tissue-resident T_reg_ cells, a special population of T_reg_ cells. Tissue-resident T_reg_ cells populate specific peripheral tissues in the body, performing non-immunological functions and being devoted to maintain tissue homeostasis and wound repair (29, 90–93). Skin is the largest organ and is home to a large proportion of the body’s tissue-resident T_reg_ cells, and skin-resident T_reg_ cells are beginning to be understood (42, 94, 95). They predominantly reside in the dermis (96) and promote wound healing and tolerance to the skin microbiota (97–99). Surprisingly, skin-resident T_reg_ cells preferentially localized to hair follicles and were most abundant in the scalp and face (with high hair follicle density), whereas T_conv_ cells displayed a more diverse distribution (42).

Furthermore, Ali et al. found that follicular T_reg_ cells of mice reside within 0–5 μm of bulge hair follicle stem cells (HFSCs) (9). We are aware that HFSCs are responsible for the cyclic proliferation of hair follicles, so do skin-resident T_reg_ cells control hair growth *via* HFSC? Inevitably, the authors further demonstrated that skin-resident T_reg_ cells with Jagged 1 expression promote HFSC proliferation and differentiation to drive HF cycling through the Notch signaling pathway. A new study reported that glucocorticoids induce glucocorticoid receptor (GR) in skin-resident T_reg_ cells to produce TGF-β3, which activates HFSCs and promotes hair regeneration (100). Given that HF is a sophisticated mini-organ of the skin and offers a “niche” for stem cells (including HFSC) (101), some investigators suggested that skin-resident T_reg_ cells might occupy a specialized HFSC niche (9, 102). Almost all T_reg_ cells (greater than 95%) in human skin have an effector memory phenotype (42); that is to say, a substantial part of T_reg_ cells in HF promote both immunosuppression and HF regeneration (**Figure 1C**). Actually, the dual role of skin-resident T_reg_ cells has been proposed by Maryanovich et al. (103). Taken together, given the HF damage is reversible in AA, we believe that protecting and restoring the functioning skin-resident T_reg_ cells might be the cornerstone of AA management.

## T_reg_ cell-based therapies: Opportunities or challenges?

To date, the majority of traditional treatments for AA are of limited efficacy (especially unsustainable remission) with a high risk of adverse effects (5, 104, 105). As an established therapy with marked curative effect, corticosteroids (including topical, intralesional, and systemic therapy) have been widely used to treat AA (106). Nevertheless, they could increase the risk of folliculitis and skin atrophy (105, 107). Methotrexate and cyclosporine may cause more severe systemic adverse effects. Allergic reactions in the form of severe dermatitis, lymphadenopathy, and urticaria are often associated with contact immunotherapy (105). Minoxidil as a monotherapy might be insufficient to achieve obvious hair regrowth (108). As for innovative strategies, Janus kinase (JAK) inhibitors appear to be most successful (109–111), while further large-scale studies are required to confirm its safety and durability (112, 113). Therefore, seeking new ways is highly desirable for patients suffering from AA. Notably, it has been widely accepted that AA is an autoimmune disease (114–117), although this concept remains a hypothesis (autoimmune target antigen has yet to be defined) and even a few people challenge it (69). AA is also related to various other autoimmune disorders (118). In fact, T_reg_ cell-based therapies have emerged as a new avenue in various human autoimmune disorders, which aim to restore balanced immunity. As T_reg_ cells are important regulators of HFSC function, T_reg_ cell-based therapies could be a type of treatment that stimulates HF regeneration. Here, we will review some existing related studies and discuss the prospects and challenges of T_reg_ cell-based AA therapies.

### Antigen-specific T_reg_ cells as “living drug”

The impact of AA is frequently underestimated and perhaps dismissed as only a “cosmetic problem” (116, 119). Coupled with the high price of cell products, little attention has, to our knowledge, been paid to cellular therapy for AA, but that could be the way to go. Previously, adoptive transfer of polyclonal T_reg_ cells has been investigated extensively; these trials fully demonstrate its feasibility and safety (120–122). However, polyclonal T_reg_ cells have been limited by the difficulty in producing quantities sufficient for clinical use and systemic immunosuppression (such as inadvertent suppression of immune responses to infection or malignancies) (68, 123), while antigen-specific T_reg_ cells could overcome these difficulties. They migrate to lesions and respond to their cognate antigen to provide more effective protection from autoimmune activity (124–127). Chimeric antigen receptor (CAR) and T-cell receptor (TCR) could redirect T_reg_ cells. Specifically, unlike TCR-T_reg_ cells, CAR-T_reg_ cells can bypass HLA restriction (128, 129) and target more flexibly (antigens recognized by CARs also include non-protein and soluble targets) (130, 131). Expectedly, CAR technology might be available to AA.

Except for graft versus host disease (GvHD, with a very clear target), selecting a suitable antigen is critical when constructing a CAR for most autoimmune diseases, although this process is time-consuming and sometimes difficult (129, 132). Owing to the great efforts of researchers, a variety of CARs have been developed and show great potential for treating various diseases in preclinical studies. Elinav et al. (133) were one of the first to develop a CAR to cure mouse colitis. MacDonald and colleagues (134) transduced human T_reg_ cells with a CAR that targets the HLA-A2 and found that A2-CAR-expressing T_reg_ cells ameliorated the progression of GvHD in a mouse model. CAR-T_reg_ cells have also been reported to be engineered with specificity for myelin oligodendrocyte glycoprotein to prevent experimental autoimmune encephalomyelitis (EAE, a model relating to multiple sclerosis in humans) (135). Excitingly, CAR-T_reg_ cells have been tested in studies on autoimmune skin disorders. A recent study described the curative effect of CAR-T_reg_ cells targeting ganglioside D3 to provide antigen-specific immune tolerance for vitiligo (136). Furthermore, the UK and US authorized the first CAR-T_reg_ cell clinical trial (STEADFAST, NCT04817774) for kidney transplant patients (137, 138).

**Figure 2:**
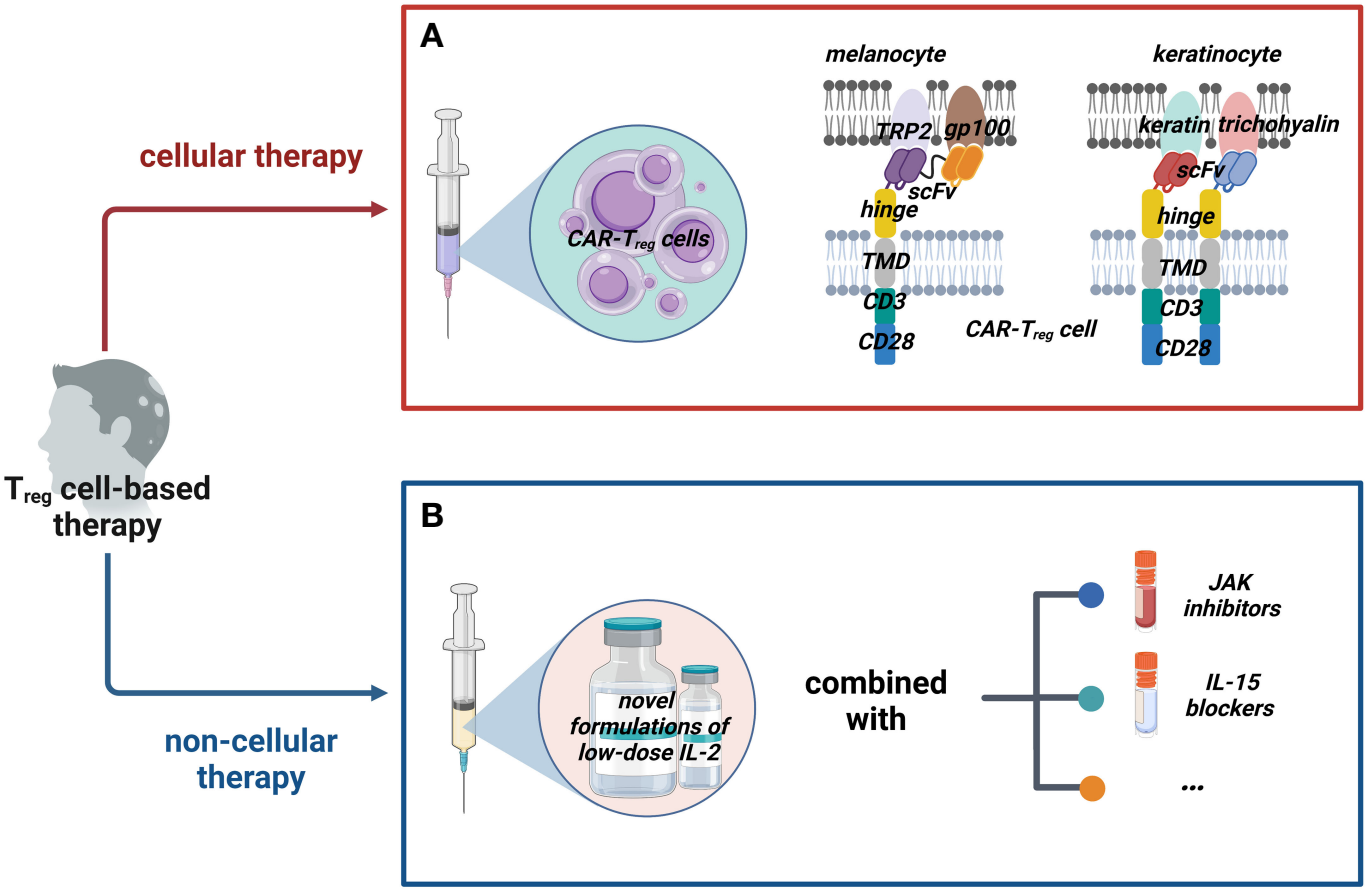
Possible T_reg_ cell-based therapies for AA. We classified possible T_reg_ cell-based therapy into two groups: cellular therapy and non-cellular therapy. **(A)** Cellular therapies such as adoptive transfer of CAR-T_reg_ cells that could target dual (or multiple) potential antigens on melanocytes and keratinocytes with appropriate costimulatory domains should be tried. **(B)** Non-cellular therapies mainly work by inducing T_reg_ cells; novel formulations of IL-2 are constantly being developed and they could be combined with other drugs such as JAK inhibitors and IL-15 blockers to enhance the efficacy. (**Figure2** created with BioRender.com).

Regrettably, owing to the hurdle that the exact HF autoantigen(s) is still disputed, there is hardly any research on the therapeutic use of CAR-T_reg_ cells for AA. Given the frequent clinical observations that AA seems to target gray hairs rather than pigmented hairs and nonpigmented hair regrowth, melanocyte antigen epitopes have long been suspected as potential targets (139–143), such as tyrosinase (TYR), tyrosinase-related protein-1 (TRP-1), TRP-2, glucoprotein100 (gp100, premelanosome protein analog), Melan-A (also known as melanoma antigen recognized by T cells 1 Leu27 analog, MART-1), and melanocortin-1-receptor (MC1R) (140, 144). Why hair loss happens rather than just graying could not be proven. That is to say, the destruction of melanocytes is only part of the story of immunoreactive HF. Our recently published review (145) argued that melanocytes might only be the initiating factor of autoimmune attack. Wang et al. (146) deduced that activated CTLs secrete multiple inflammatory cytokines harmful to keratinocytes even without direct cell–cell contact. IFN-γ upregulates MHC I and MHC II molecules in HF, potentially triggering antigen presentation in other cell populations. Many researchers have discovered keratinocyte-derived autoantigens, such as trichohyalin (expressed in the growing HF’s inner root sheath) and keratin (expressed in the anagen HF pre-cortical zone) (146–148). Although the authors speculated that these proteins were the major autoantigens in human AA, definitive evidence is lacking. We think that the above elusive melanocyte- and keratinocyte-targeting antigens should be used to exploit CAR-T_reg_ cells and initially investigate their effects on AA in animal models because of their great significance: some autoantigens of AA could be confirmed on the one hand, and the effectiveness of CAR-T_reg_ cells could be examined on the other hand (**Figure 2A**).

Specifically, for CARs, the following aspects need to be considered. In general, increasing CAR’s specificity could enhance T_reg_ cells’ effect. As a result, most people focused only on mono-specific CAR-T_reg_ cells, which sometimes fall short of our expectations. For example, some scientists generated a CAR against human insulin and found that the T_reg_ cells were suppressive and long-lived, but did not prevent type 1 diabetes (T1D) in mice (149). Mohseni et al. provided a new perspective in their review (138); the methods of implementing universal recognition of CAR-T_reg_ cells were summarized: mixing CAR-T_reg_ cells against different antigens, constructing a T_reg_ cell with several CARs or one multi-specific CAR against different antigens, and building UniCAR-T_reg_ cells. Perhaps, these novel CAR-T_reg_ cells targeting multiple antigens in HF will make a difference, which awaits confirmation. It should be pointed out that choosing the appropriate costimulatory domain is also crucial. The costimulatory domains of CAR-T_reg_ cells currently used are the same as those used in conventional CAR-T cells, but they could result in different effector functions (150). A typical example is that 4-1BB may reduce the suppressive function of CAR-T_reg_ cells (151, 152). This means that CD28 instead of 4-1BB should be considered the costimulatory domain of CAR-T_reg_ cells for AA. The relevant mechanism needs to be better understood; perhaps the presence of one or several costimulatory domains contributes to the optimal function in AA.

All in all, the identification of the specific autoantigen(s) is a prerequisite for targeted therapy. Only our comprehensive knowledge about the target antigens in AA could open the door to antigen-specific T_reg_ cell therapy for it.

### Induction of T_reg_ cells *in vivo*: Low-dose IL-2

As an alternative strategy for T_reg_ cell transfer, some agents could also increase T_reg_ cell numbers and support T_reg_ cell function. Compared to transferring living cells, this approach will bring two obvious advantages: improved cost-effectiveness and lower risk for adverse events. In a deeper sense, for AA, these therapeutics may induce naïve T cells to develop into Foxp3^+^ T_reg_ cells to re-establish immune homeostasis and activate skin-resident T_reg_ cells to promote HF regeneration in the lesional scalp.

Nowadays, there are lots of existing therapeutic agents that target T_reg_ cells, such as IL-2, TGF-β, rapamycin, or CTLA-4Ig. Foxp3 expression and histone acetylation are induced by TGF-signaling (153–155). The PI3K–AKT–mTOR axis activation is a crucial negative regulator of T_reg_ cells (156, 157), and rapamycin could promote the T_reg_ cell expansion (158–161). IL-2 is the most widely used during the last couple of years. T_reg_ cell activation and proliferation highly depend on IL-2 production, mainly by T_conv_ cells, a key mechanism to prevent T cells’ overexpansion (162–164). T_conv_ cells can be induced to express Foxp3 in the presence of IL-2 (43, 165), whereas anti-IL-2 antibodies could impair T_reg_ cells (166). *In vivo*, IL-2 is crucial for T_reg_ cell survival, proliferation, and stability (167–169), suggesting that IL-2 is an important agent for regulating T_reg_ cells.

Encouragingly, some promising data have been achieved with IL-2 to treat AA. Subcutaneous injection of low-dose recombinant IL-2 allowed the recruitment of T_reg_ cells inside the lesional scalp skin in four of five patients with severe AA refractory to prior systemic therapies, as shown by Castela et al. (170), but the remaining patient did not show improvement. In addition to AA, low-dose IL-2 has positively affected other autoimmune diseases, including systemic lupus erythematosus, T1D, hepatitis C virus-induced vasculitis, and immune thrombocytopenia (171–175). Theoretically speaking, T_reg_ cells can efficiently compete for a limited amount of IL-2 with effector cells because the former expresses a higher-affinity IL-2 receptor, and is sensitive to low levels of IL-2 (56, 176, 177). However, the dose of IL-2 should be treated with caution, because its slight increase could activate conventional memory T cells and NK cells, namely, off-target complications (172, 178). Patients must be administered IL-2 regularly and at short intervals because of its short half-life (179). Consequently, the IL-2 therapy protocol in AA should be optimized in Castela et al.’s study. To address the drawbacks of utilizing pure IL-2, many investigators have improved IL-2 therapy. For example, controlled release formulations (IL-2/TGF-β1/rapamycin) (180), dual-acting cytokine fusion protein (IL2-EHD2-sc-mTNFR2) (181), long-lived IL-2 mutein [IgG-(IL-2N88D)_2_] (182), and IL-2/UFKA-20 complex (183) have been developed successively. These novel formulations achieved superior T_reg_-expanding properties and selectivity.

Recently, some negative results attracted our attention. In 2020, 43 adult patients with severe AA completed a multicentric randomized placebo-controlled trial with a 52-week follow-up period (184). Unfortunately, despite significantly increasing peripheral T_reg_ cells due to IL-2 therapy, these patients failed to achieve noticeable hair regrowth. The authors hypothesized that the limitation of increasing T_reg_ cells only to the naïve subset (without skin-homing capabilities) is partly responsible for poor efficacy. Not coincidentally, another study reports a similar phenomenon: the T_reg_ cells of mice injected with the IL-2 cytokine antibody complex (consisting of human IL-2, anti-hIL-2 antibody, and mouse IL-2 Fc) were 8–10 times higher than those of the control group. However, this administration cannot reverse AA on the established mouse model (185). As the authors note, the treatment of IL-2 confronted the problem of whether combination therapy is needed. Indeed, co-medication has shown promise in other autoimmune diseases (186). The IL-2 therapy combined with the application of JAK inhibitors (187, 188), blocking IL-15 transpresentation (189), and more would be worth investigating in future studies (**Figure 2B**). Additionally, Ferreira et al. (131) suggested that IL-2 therapy could be combined with cellular therapy of T_reg_ cells. Three clinical trials are currently testing whether this hypothesis could increase efficacy in T1D (NCT02772679), steroid-refractory chronic GvHD (NCT01937468), and amyotrophic lateral sclerosis (NCT03241784), which could also provide us some insight.

## Conclusions

Autoimmune disorders go together with the impairment of T_reg_ cells. Suppressing abnormal autoimmunity by boosting T_reg_ cells in patients is a rational approach. An increasing number of clinical trials on them are being conducted to evaluate safety (including possible side effects) and efficacy. Although the pathogenesis of AA remains incompletely understood, T_reg_ cells have been considered to be involved. It should be pointed out that many questions need to be answered: “what is the exact autoantigen of AA?”, “do autoantigen responses play a primary role in AA pathobiology?”, “are the defects of T_reg_ cell the cause or effect of HF-IP collapse?”, and so on. However, this does not deny the strong link between T_reg_ cells and AA. T_reg_ cells play a major role in establishing and maintaining local self-tolerance as HF-IP guardians. Moreover, recent studies on tissue-resident T_reg_ cells revealed their unique biological functions. In particular, T_reg_ cells located in the HFSC niche could promote hair follicle regeneration, allowing us to better understand the greater potential of T_reg_ cell-based therapies in AA than other autoimmune skin diseases. Owing to its unique pathophysiology, AA is difficult to manage medically. Despite the promising drug JAK inhibitors, long-term efficacy is still limited. Thus, there is no harm in conducting a T_reg_ cell-based therapy investigation; this work could guide clinical practice. We believe that T_reg_ cell-based therapy is the next logical step for AA management, which ultimately may improve patient outcomes.

## Author contributions

SW, CG and XS participated in manuscript writing. CG, XS, WX and BX collected the literature and provided general idea. CG contributed to manuscript editing. All authors contributed to the article and approved the submitted version.
